# Rainfall drives variation in rates of change in intrinsic water use efficiency of tropical forests

**DOI:** 10.1038/s41467-019-11679-8

**Published:** 2019-08-14

**Authors:** Mark A. Adams, Thomas N. Buckley, Tarryn L. Turnbull

**Affiliations:** 1 Department of Chemistry and Biotechnology, Faculty of Science, Engineering and Technology, Swinburne University of Technology, Melbourne, VIC Australia; 2 School of Life and Environmental Sciences, University of Sydney, Sydney, NSW Australia; 3 Department of Plant Sciences, College of Agricultural and Environmental Sciences, University of California, Davis, CA USA

**Keywords:** Atmospheric chemistry, Plant physiology, Carbon cycle, Carbon cycle, Ecophysiology

## Abstract

Rates of change in intrinsic water use efficiency (*W*) of trees relative to those in atmospheric [CO_2_] (*c*_a_) have been mostly assessed via short-term studies (e.g., leaf analysis, flux analysis) and/or step increases in *c*_a_ (e.g., FACE studies). Here we use compiled data for abundances of carbon isotopes in tree stems to show that on decadal scales, rates of change (*dW/dc*_a_) vary with location and rainfall within the global tropics. For the period 1915–1995, and including corrections for mesophyll conductance and photorespiration, *dW/dc*_a_ for drier tropical forests (receiving ~ 1000 mm rainfall) were at least twice that of the wettest (receiving ~ 4000 mm). The data also empirically confirm theorized roles of tropical forests in changes in atmospheric ^13^C/^12^C ratios (the ^13^C Suess Effect). Further formal analysis of geographic variation in decade-to-century scale *dW/dc*_a_ will be needed to refine current models that predict increases in carbon uptake by forests without hydrological cost.

## Abstract (web summary)

How the water use efficiency of trees changes with atmospheric CO_2_ variations has mostly been studied on short time scales. Here, a newly compiled data set covering 1915 to 1995 shows how rates of change in water use efficiency vary with location and rainfall over the global tropics on a decadal scale.

## Introduction

Rate(s) at which plant processes adjust, acclimate and adapt to rising atmospheric [CO_2_] (*c*_a_), especially processes that govern exchanges of carbon and water with the atmosphere (and their roles in ‘physiological forcing’ of climates^1–3^), have profound global implications for policy, practice and predictive models. This significance has been recognised by major research infrastructure (e.g., in free-air carbon enrichment (FACE) studies^4^), decades-long monitoring programs of atmospheric chemistry (e.g.,^5^) and a vast array of modelling studies (e.g. ref. ^6^).

For more than 30 years, theory has suggested rising *c*_a_ should increase the intrinsic water-use efficiency (*W*) of plants^7^. There is also an extraordinary volume of empirical research on *W* (and its components-photosynthetic carbon fixation, *A*, and stomatal conductance, *g*_s_) across ecosystems. For temperate and boreal forests, the theory of rising *W* with *c*_a_ is thus backed by a large body of work (e.g., refs. ^8–13^), and increases in *W* of trees are amongst the most common of global responses to rising *c*_a_, albeit with exceptions. Evidence of increases in *W* comes from multiple sources including large-scale flux networks^8,9^, tree ring (or ring proxy) isotope series^10^, catchment-scale studies^11^, model-data fusions^11–13^ and hundreds of leaf-scale analyses (e.g.,^14^), some based on historic herbarium samples^15^. However, and in contrast to the general case that *W* should rise with *c*_a_, the long-term rate at which *W* has been changing has not been rigorously examined across ecosystems or regions or climates.

Many individual studies note that *W* has seemingly increased more quickly since the 1960s, in concert with the faster rate of increase in *c*_a_, but formal examinations are largely restricted to leaf-level and relatively short-term studies^4,16^ (see also ref. ^11^ for a 28-year study). ^13^C/^12^C ratios of cellulose derived from annual tree rings (or from otherwise age-identified wood) provide a time-integrated measure of *W* that can be extended to hundreds of years in the case of long-lived trees, and used as an important complement and contrast to shorter-term (often leaf-level) data^17^. Such data have been widely used to test models of feedbacks amongst the biosphere, atmosphere and climate. Some of the more significant constraints to its interpretation, such as potential confounding (ontogenic) effects of tree age and size, have recently been characterised^18^.

We asked the broad question: what are the rates of change in *W* of tropical forests? We followed recent suggestions^19^ in focusing on isotope series (time series of abundances of stable isotopes of C, as captured in stemwood) as a means of improving our ability to predict responses of forests to global change. Our formal hypotheses were that in the long term, *W* increases with *c*_a_, and *dW/dt* or *dW/dc*_a_ will depend on climate. We used tropical forests to test these hypotheses, since previous theoretical predictions^20,21^, and medium-term (<30 years) catchment studies^11^, have explicitly supported our first hypothesis, albeit over shorter time scales. The significance of our hypotheses was recently demonstrated in a modelling study^22^, which suggested that local changes in the rate at which *W* adjusts to *c*_a_ (physiological forcing) are responsible for the majority of precipitation change above tropical forests. Isotope series data revealed that rainfall is a significant determinant of long-term (80 year) *dW/dc*_a_, as is nitrogen-fixing capability (legume vs. non-legume).

## Results

### Data availability and preliminary analysis

From a global search (see the Methods section), we compiled two data sets. Data Set 1 was composed of all available isotope series^23–29^ that spanned the period 1915–1995, excluding data from heavily modified sites. Data Set 2 comprised all other isotope series for the tropics^30–38^. Using the traditional calculation (see the Methods section), analysis of all available data (Data Set 1 + Data Set 2, Supplementary Table 1, Supplementary Fig. 1b) reveals that for 32 of 42 isotope series, *W* had a significant positive relationship with *c*_a_ (as it does in temperate and boreal forests^8–10,12,13^). For eight, mostly short-term series, there was no relationship, and two series showed negative relationships. It is instructive that one such negative relationship between *W* and *c*_a_^34^ was based on a short-term study (1925–1938) during the Great Depression (1929–1939)—a period of exceptionally slow annual rates of increase in *c*_a_. When compared, mean *W* for both data sets was very similar (and not significantly different) for a period when nearly all isotope series overlapped (1990–1995; Supplementary Fig. 1c), notwithstanding some isotope series in Data Set 2 showing fast rates of change in *W* with *c*_a_.

### Analysis of long-term data

**Fig. 1:**
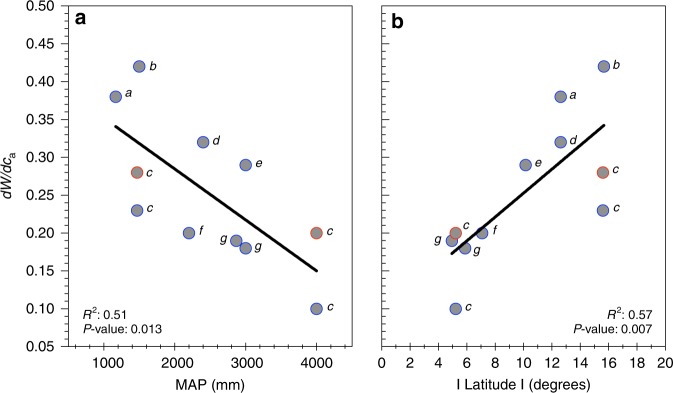
Rainfall and latitude influences on change in *W* per unit *c*_a_. **a** The relationship of *dW/dc*_a_ (change in intrinsic water-use efficiency (*W*) per unit atmospheric [CO_2_] (*c*_a_); as derived from tree rings) to mean annual precipitation. **b** Relationship of *dW/dc*_a_ to absolute latitude. The data are site averages of long-term isotope series (each spanning at least the period 1915–1995) for tropical biomes. Points marked in red in both (**a**) and (**b**) are the data for legumes. For both (**a**) and (**b**), data sources are*:* (a) Wils et al.^24^, (b) Nock et al.^27^, (c) van der Sleen et al.^23^, (d) Ballantyne et al.^25^, (e) Hietz et al.^29^, (f) Schollaen et al.^28^, (g) Loader et al.^26^

**Fig. 2:**
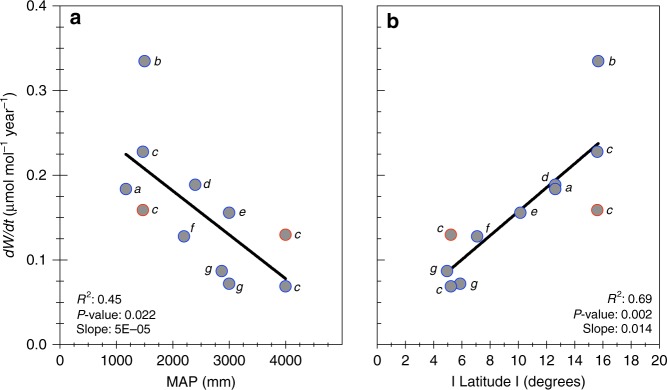
Rainfall and latitude influences on change in *W* per unit time. The relationship of *dW*/*dt* (change in intrinsic water-use efficiency (*W*) per year; as derived from tree rings) and mean annual precipitation (**a**), and absolute latitude (**b**). The data are site averages of long-term isotope series (each spanning at least the period 1915–1995) for tropical biomes. Points marked in red in both (**a**) and (**b**) are legumes. For both (**a**) and (**b**), data sources are: (a) Wils et al.^24^, (b) Nock et al.^27^, (c) van der Sleen et al.^23^, (d) Ballantyne et al.^25^, (e) Hietz et al.^29^, (f) Schollaen et al.^28^, (g) Loader et al.^26^

We subjected all available long-term isotope series (Data Set 1) to more detailed analysis. Positive relationships of *W* with *c*_a_ were just as clear when data were aggregated by site (see Table 1; Supplementary Fig. 2). All long-term relationships between *c*_a_ and *W* were linear and highly significant (Table 1; *P* < 0.0001)), indicating relative invariance in the ratio of intercellular (*c*_i_) to *c*_a_ (as *W* *=* *c*_a_(1 − *c*_i_/*c*_a_)/1.6; see Eq. (3) in the Methods section). Across the tropics, *W* of canopy dominants has increased at different rates in response to increasing *c*_a_, with fourfold differences amongst sites in *dW/dc*_a_ or *dW/dt* (Fig. 1a, b). Perhaps surprisingly, mean annual precipitation (MAP) has alone accounted for half of the site-to-site variation in *dW/dc*_a_ (Fig. 1a). When expressed on an annual (time) basis, *dW/dt* declined by 0.05 μmol mol^−1^ year^−1^ for each 1000 -mm increase in rainfall (Fig. 2a). In other words, over the course of the last century the *W* of trees at the driest included site increased by at least 15 μmol mol^−1^ more than of trees at the wettest. Both *dW/dc*_a_ and *dW/dt* were even more strongly related to latitude (Fig. 1b, 2b), and were greatest for systems distinguished by a distinct dry season (see also ref. ^39^). The ratio of MAP to potential evapotranspiration (PET) was also significantly related to *dW/dc*_a_ (see Supplementary Fig. 3a), again explaining ~ 50% of the variation. Mean annual temperature was not related to *dW/dc*_a_ (Supplementary Fig. 3b).

**Table 1:** Significant bivariate relationships between inherent water-use efficiency (*W* μmol mol^−1^) and atmospheric [CO_2_] (*c*_a_ ppm) for canopy-dominant trees in tropical biomes (minimum period = 1915–1995)

Country, rainfall, legume status	No. of species	No. of trees sampled	Data source	Equation	*R* ^2^	*P-*value
Ethiopia, 1170 mm, non-legume	1	5	Wils et al.^24^	*W* = −18.4 + 0.38*c*_a_	0.91	0.000
Thailand, 1500 mm, non-legume	2	27	Nock et al.^27^	*W* = −78.9 + 0.42*c*_a_	0.99	0.000
Thailand, 1470 mm, legume	1	82	van der Sleen et al.^23^	*W* = 1.8 + 0.28*c*_a_	0.51	0.000
				*W* = −440 + 92ln(*c*_a_)	0.50	0.000
				*W* = 17 + 0.19*c*_a_ + 0.0001*c*_a_^2^	0.51	0.000
				*W* = 0.34*c*_a_^0.97^	0.50	0.000
Thailand, 1470 mm, non-legume	3	179	van der Sleen et al.^23^	*W* = 3.7 + 0.23*c*_a_	0.32	0.000
				*W* = −390 + 81ln(*c*_a_)	0.33	0.000
				*W* = −154 + 1.2*c*_a_ − 0.001*c*_a_^2^	0.33	0.000
				*W* = 0.28 *c*_a_^0.98^	0.31	0.000
Peru, 2400 mm, non-legume	1	1	Ballantyne et al.^25^	*W* = −34 + 0.32*c*_a_	0.79	0.000
Indonesia, 2200 mm, non-legume	1	16	Schollaen et al.^28^	*W* = 8.9 + 0.20*c*_a_	0.72	0.000
Borneo, 2870 mm, non-legume	2	2	Loader et al.^26^	*W* = 2.2 + 0.18*c*_a_	0.77	0.000
Borneo, 3000 mm, non-legume	1	2	Loader et al.^26^	*W* = −4.4 + 0.18*c*_a_	0.72	0.000
Brazil, 3000 mm, non-legume	2	53	Hietz et al.^29^	*W* = −22.6 + 0.3*c*_a_	0.98	0.000
Cameroon, 4000 mm, legume	3	268	van der Sleen et al.^23^	*W* = 13.8 + 0.20*c*_a_	0.24	0.000
				*W* = −302 + 66ln(*c*_a_)	0.25	0.000
				*W* = −354 + 2.43*c*_a_ − 0.003*c*_a_^2^	0.29	0.000
				*W* = 0.57*c*_a_^0.85^	0.24	0.000
Cameroon, 4000 mm, non-legume	1	94	van der Sleen et al.^23^	*W* = 36.3 + 0.11*c*_a_	0.19	0.000
				*W* = −130 + 35ln(*c*_a_)	0.19	0.000
				*W* = −35 + 0.5*c*_a_ − 0.001*c*_a_^2^	0.19	0.000
				*W* = 4.3*c*_a_^0.48^	0.16	0.000

Relationships are shown for each site and study. The data were pooled (i.e., across species) for each site according to their nitrogen-fixing status (non-legume or legume)

**Fig. 3:**
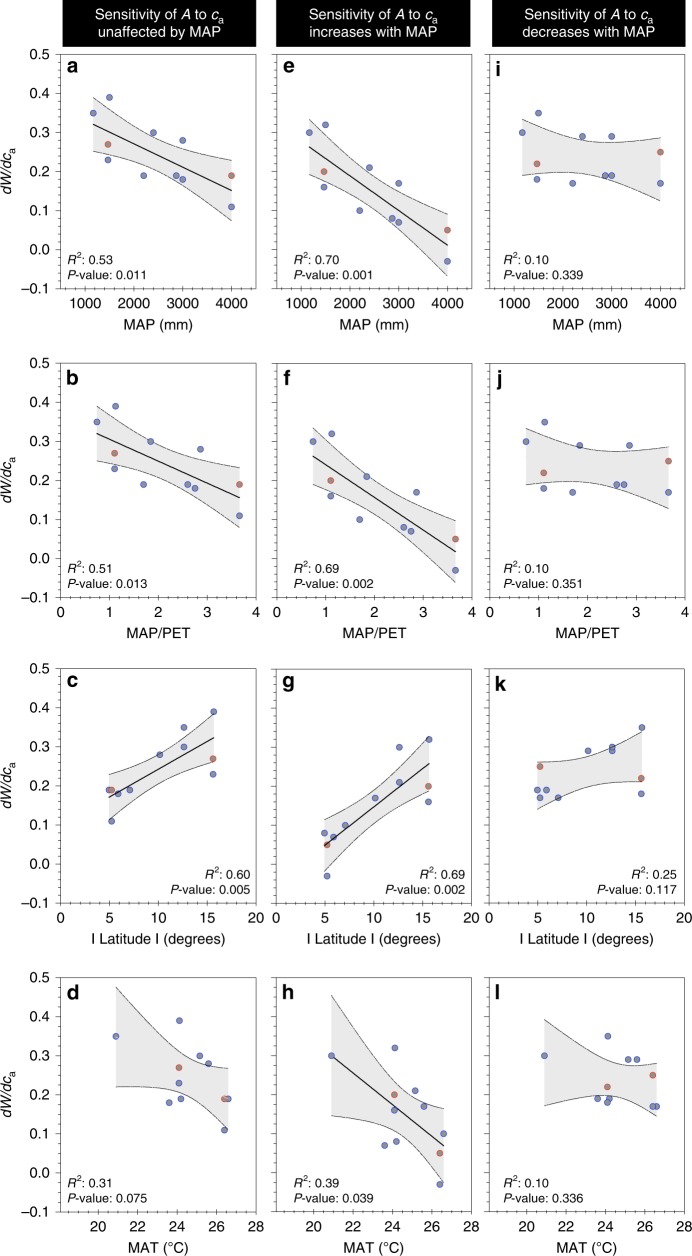
Modelled effects of physiology on rates of change in *W*. Effects of methods for computing intrinsic water-use efficiency (*W*), and modelled sensitivity to mean annual precipitation (MAP) of the *A/c*_a_ response to *c*_a_ (where *A* is net photosynthesis and *c*_a_ is atmospheric [CO_2_]) for relationships of *dW*/*dc*_a_ (change in *W* per unit *c*_a_) to: MAP, the ratio of MAP to potential evapotranspiration, absolute latitude and mean annual temperature (MAT). **a**–**d** *W* includes terms to account for mesophyll conductance and photorespiration. **e**–**h** as for **a**–**d** but *A/c*_a_ response to *c*_a_ increases with MAP, (**i**–**l**) as for **a**–**d** but *A/c*_a_ response to *c*_a_ decreases as MAP increases. The data are site averages from sources as described in Figs. 1 and 2. See the Methods section for further detail

When we followed Keeling et al.^5^ in adopting a more comprehensive approach to calculating *W*, and included potential effects of mesophyll conductance and photorespiration on isotope discrimination, there was little change in relationships of *dW/dc*_a_ to MAP, MAP/PET and latitude (Fig. 3a–c). Similarly, we modelled impacts of potential changes in the ratio *A*/*c*_a_ (where *A* is net photosynthesis) with increasing *c*_a_ on the relationship of *dW/dc*_a_ to MAP and latitude, in order to test if observed relationships with rainfall might be due to other influences over the past 100 or so years. As one limit, we assumed that *A*/*c*_a_ remains constant (i.e., *A* increases in proportion to *c*_a_). As the other, we assumed that *A* remains constant regardless of any rise in *c*_a_. If MAP had no effect on the sensitivity of *A* to *c*_a_ then patterns shown in Figs. 1 and 2 are little changed (Fig. 3a–c). If *A* changed in proportion to *c*_a_ at the lowest rainfall, but became increasingly insensitive to *c*_a_ as rainfall increased (and was constant at the highest rainfall), then relationships were much weakened (Fig. 3i–k). Finally, if the sensitivity of *A* to *c*_a_ increased with rainfall, then relationships strengthened (Fig. 3e–g). While arguments can be made for all scenarios, the extreme case shown in Fig. 3i–k is highly unlikely, and recent evidence from tropical forests^11^ suggests the scenario in Fig. 3a–c is most likely.

Growth of individual species responded variably to rising *c*_a_ (Supplementary Table 1), although the magnitude of responses depended on both the period of measurement and site/climate (see also Supplementary Fig. 1b). Most species showed no distinct growth response to rising *c*_a_, in contrast to positive responses of *W* (Supplementary Table 1). We also examined other possible influences on *W*. Clear patterns of decreasing *dW/dc*_a_ (Fig. 1) or *dW/dt* (Fig. 2) with increasing rainfall, are the opposite of what might be expected if tree growth, rather than *c*_a_, were driving changes in *W* (see ref. ^18^).

The novel approach and related sampling adopted by van der Sleen et al.^23^ accounts for potential bias due to effects of ontogenic development on *W*. Nonetheless, the van der Sleen et al.^23^ data integrate well with all other available data for the tropics (e.g., Figs. 1–3), including data from single-tree studies.

Nutrient availability is another, frequently suggested, non-climatic constraint to tree responses to rising *c*_a_. The van der Sleen et al. data^23^ contained both legumes and non-legumes, which we have partitioned accordingly. Legumes (Supplementary Fig. 4) maintained significantly greater *W* than non-legumes, over the full period for which records were obtained (a difference of 7–8 μmol mol^−1^), in agreement with a recent synthesis of leaf-level data^40^. In all analyses shown in Figs. 1–3, and Supplementary Figs. 2 and 3, data for legumes are identified separately.

## Discussion

Collectively, the data compiled here represent tens of thousands of individual measurements of abundance of stable isotopes of carbon in wood samples from a broad range of climates and tree species. Represented ecosystems include monsoonal conifer forests in the Ethiopian highlands (*Juniperus*), Congo basin rainforests in Cameroon (*Daniellia, Terminalia* and *Brachystegia*), lowland dipterocarp rainforests in Indonesia and in Borneo (*Tectona, Shorea* and *Eusideroxylon*) and savannas and forests of Thailand (*Melia, Toona* and *Chukrasia*) and Brazil (*Swietenia, Cedrela* and *Sweetia*). The data show that long-term rates of change in *W* for trees from tropical biomes (Supplementary Table 1; range 0.10–0.43 μmol mol^−1^ year^−1^) are broadly comparable with trees from boreal (0.22 μmol mol^−1^ year^−1^), semi-arid (0.17 μmol mol^−1^ year^−1^) and temperate (0.28 μmol mol^−1^ year^−1^) forests in North America^9^ and Europe^8,12^.

Keeling et al.^5^ recently calculated that to account for the changing relative abundances of ^13^C vs. ^12^C in atmospheric CO_2_ (the ^13^C-Suess effect), there must have been a ~20% increase in *W* across the globe over the 40 years period 1975–2015, and that a significant proportion of the increase must have been due to tropical forests. The data compiled here provide an empirical confirmation of that calculation for tropical forests (Supplementary Table 1). As far as we can ascertain, our analysis is also the first evidence of rainfall-driven and systematic variation in long-term rates of change in water-use efficiency for any global forest biome. Current models of global patterns in *W* do not account for this variation (e.g., ref. ^6^).

Shorter term, more recent studies are an interesting contrast to the long-term patterns that are the focus here. For example, trees used by Nock et al.^27^ spanned generally shorter time periods (88 years) than other long-term isotope series (see Figs. 1–3), and their data are somewhat of an outlier. Greater *dW/dc*_a_ (Fig. 1a, b), and greater *dW/dt* recorded by Nock et al.^27^, is at least partially due to faster rates-of-increase in *c*_a_ in recent decades. Other shorter-term (mostly post 1960) isotope series for the tropics (see Supplementary Table 1, Supplementary Fig. 1 and refs. ^30–38^), as well as leaf-level studies^14,15^ strongly support shown long-term patterns (including the influence of rainfall; see ref. ^41^ for a summary of effects of rainfall on *W* within individual studies of both ring-forming and ringless trees).

Obviously, rainfall alone does not define water availability to trees. In the tropics especially, seasonal distributions of rainfall and evaporative demand ensure that soil water storage plays a major role in year-round water availability, which in turn is reflected in seasonal variation in photosynthetic productivity of tropical forests^39^. Broadly speaking, tropical regions with pronounced dry seasons (typically savannah systems) can also be distinguished by seasonal changes in leaf area and transpiration from regions that have more uniform rainfall^39^. Nonetheless, rainfall is a strong predictor of *dW/dc*_a_ across long-term studies (e.g., Fig. 1), as well as within shorter-term and individual studies^41^ of tropical forests.

Donohue et al.^42^ used modelling and FACE data to argue that rates of carbon fixation have broadly increased with *c*_a_. They also showed that disturbance plays a significant role in the reliability of modelled predictions of vegetation responses to *c*_a_. Our analysis supports a conclusion that rates of carbon fixation have continued to rise with *c*_a_ in tropical forests, irrespective of water availability. Disturbances (e.g., fires, floods, hurricanes) are frequently associated with major changes in nutrient and water availability. Evidence (Supplementary Fig. 4) of significant influence of nitrogen fixation on *W* (but not on *dW/dc*_a_), as well as the enhanced abundance of legumes after disturbance, points to the need for a stronger focus on regenerating forest ecosystems as a means of disentangling more proximal (e.g., nutrient availability) and distal (e.g., disturbance) influences on *dW/dc*_a_.

As reviewed a decade ago^43^, the significance of tropical forests to global carbon and hydrological cycles can scarcely be overstated. That significance has prompted many calls for increased research, for example into the notoriously unknown acclimation responses of plant physiology to rising *c*_a_^21,44^. Large year-to-year variation in climatic conditions, phenology and disturbance can easily render tenuous any conclusions based on shorter-term experimentation and observation, as well as models built on such foundations. Long-term rates of change in *W* shown here, based on integration of climatic and atmospheric information in tree stems, remain one of the best available means of validating and improving models for the tropics. Recorded *dW/dc*_a_ (and *dW/dt*) will also help guide efforts to predict future changes in *W*, at least for the tropical biome, over coming decades. We endorse the call^19,41^ for greatly increased availability of isotope series data (as collated here) for tropical forests owing to its scientific significance. However, we immediately recognise that developing such an increased availability of data (and being able to reliably apply statistical approaches such as meta-analysis) will likely take many years, if not decades.

A key unknown in the broad field of global climate change is how quickly forests adjust/acclimate/adapt to changing atmospheric and climatic conditions. Evidence here for the tropics suggests that while *W* has increased with *c*_a_, differences in water availability at any given site determined large shifts/variation in the rate of change (*dW*/*dc*_a_). This helps constrain thinking and models directed towards resolving drivers of changing rainfall in the tropics (e.g., ref. ^22^), and elsewhere. For the tropics, recent evidence^45–47^ also suggests a slowing of growth and weakening of the tropical forest sink for carbon. At some point, carbon uptake by forests can no longer increase without commensurate increases in availability of water and nutrients. Analysis of water limitation and drought effects on physiological performance^48,49^ emphasises that at least parts of the tropics may be approaching limits to the rate of change in *W* with *c*_a_ (see ref. ^3^; Supplementary Fig. 4)−a phenomenon for which there is also evidence from temperate forests in Europe^50^. The roles of water and nutrient availability as regulators of *dW/dc*_a_ need further elucidation.

## Methods

### Identification of data

We identified relevant literature by screening the Web of Science and Google Scholar search engines for keywords: dendrochron*, cellulose, tree ring, carbon isotope discrimination, δ^13^C, WUE, water-use efficiency, tropic* and also included relevant citations documented within these literature. We only accepted literature for forests classified as tropical (Köppen Climate Classification A). We identified 42 isotope series from 16 published studies that could provide estimates of *W* for tropical forests. The majority of isotope series were of short duration. Two were derived from sites that had been subject to heavy modification (clearing, fertiliser use etc.), and these were not used in this study.

Latitude and longitude of each site was used to identify MAP (mm), mean annual potential evaporation (PET), MAT, (Climatic Research Unit, University of East Anglia) and confirm sites were of Köppen classification A (tropical/megathermal). Constant vapour pressure difference between air and intercellular airspace would ensure trends in *W* derived from wood cellulose represent trends in water-use efficiency^23^, and previous studies show vapour pressure deficits (VPD) for tropical biomes (and elsewhere) have changed little during the past 150 years^51^.

### Data inclusion and testing

Our analysis includes the multi-species, multi-site data set of van der Sleen et al.^23^, as well as isotope series from studies that encompass smaller numbers of sites and species (see Table 1; Supplementary Table 1, for details); including some single-tree studies. The data represent deciduous, evergreen and semi-deciduous trees, and also represents both ring-forming and ringless trees. The longest isotope series was almost 250 years, and the shortest was less than a decade (See Supplementary Fig. 1b).

In addition, the novel method of van der Sleen et al.^23^ (sampled wood located at a nominated diameter within each core from large numbers of trees rather than sampling all adjacent rings within a few trees; each value for *W* corresponds with an individual tree of a given age/size) allowed us to test for differences amongst sympatric legumes and non-legumes. We excluded data from their Bolivian site, owing to the unusual, and highly localised, *terra preta* soils (nutrient-enriched by centuries of human habitation).

We tested for the potentially confounding effects of century-scale changes in photosynthesis, mesophyll conductance and photorespiration (see below). Likewise, we tested if climate-related biases in photosynthetic responses to *c*_a_, influenced patterns in *W* (see below). We based our analysis on cellulose-δ^13^C derived from rings (or from wood of otherwise identified age) of trees from tropical forests around the world (Supplementary Fig. 1a).

### Statistical approach and data quality

Our investigation was focused on exploring rates of change in *W* per unit change in atmospheric CO_2_ (*dW/dc*_a_), and effects of climate on *dW/dc*_a_. We considered a range of statistical approaches. Our research questions and available data were not well suited to meta-analytical tools (inappropriate use of these has been recently summarised^52^). Instead, and as far as possible, we present data as originally reported in each individual study (e.g., see Supplementary Figs. 2,  4). We used regression analysis to examine non-*c*_a_ influences (e.g., climate, location) on *dW/dc*_a_. Originally reported data for isotope abundances were derived from Tables and Figures, or obtained from the authors. Isotope series data are most commonly recorded in conjunction with wood age (as identified via rings or other means), measured in years, and we used all data as reported. All calculations of long-term *dW/dc*_a_ were based on the entire reported isotope series (provided the series met our criterion of spanning at least 1915–1995).

We created two data sets: Data Set 1 comprised 18 long-term isotope series (Supplementary Table 1) that encompassed the period 1915–1995 for 15 species from 9 different, largely undisturbed sites (two sites provided data for both legumes and non-legumes); Data Set 2 comprised 22 isotope series from a further 18 species and 16 undisturbed sites. Data Set 2 contained many studies that overlapped Data Set 1 for the 5-year period 1990–1995 (Supplementary Fig. 1).

Data Set 1 combined data for 720 individual trees across the major tropical forest biomes of Asia, Africa and South America (Table 1). While our focus was long-term rates of change in *W* (Data Set 1), we also sought to ensure that the long-term data reflected the broader data for tropical forests by comparing Data Sets 1 and 2 over the period 1990–1995 (for which there were the greatest number of overlapping isotope series).

### Testing for other influences on *c*_i_/*c*_a_

We used the standard approach^53^ (as used by all tree ring studies reported here^23–29^^,^^30–38^) to calculate Δ from the δ^13^C of wood cellulose. We then used four approaches to calculate *c*_i_/*c*_a_ from Δ:

(Approach ^#^1) Ignoring effects of mesophyll conductance and photorespiration.

This is the ‘standard’ and most widely used approach (see also ref. ^53^, as follows: Eq. (1).

1 $$c_{\mathrm{i}}{\mathrm{/}}c_{\mathrm{a}}= (\Delta-a)/\left( {b-a} \right)$$

with *b* = 27‰ and *a* = 4.4‰.

This approach was used for all calculations apart from those presented in Fig. 3.

(Approaches ^#^2–4) Accounting for effects of mesophyll conductance (*g*_m_) and photorespiration.

In these three approaches we used the same formulation as Keeling et al.^5^: Eq. (2).

2 $$c_i/c_{\mathrm{a}} = (\Delta-a + \left( {b-a_{\mathrm{m}}} \right)\left( {A/c_{\mathrm{a}}} \right)/g_{\mathrm{m}} + f\Gamma_ \ast /c_{\mathrm{a}})/\left( {b-a} \right)$$

with *b* = 30‰, *a* = 4.4‰, *a*_m _= 1.8‰, *f* = 12‰, *g*_m _= 0.2 mol m^−2^ s^−1^ and Γ_*_ (photorespiratory CO_2_ compensation point) = 43 ppm.

The ratio *A*/*c*_a_ (where *A* is net photosynthesis) typically declines as *c*_a_ increases, which can be modelled as *A*/*c*_a _= (*A*/*c*_a_)_280_·(*c*_a_/280)^*β*^, where (*A*/*c*_a_)_280_ is *A*/*c*_a_ at *c*_a _= 280 ppm, *β* = ln(DR)/ln(2) – 1 and DR is the doubling ratio (i.e., DR = 1.45, if *A* increases by 45% with doubling of *c*_a_ from 280 to 560 ppm).

For Approach ^#^2, we assumed DR = 1.45, following Keeling et al.^5^. If we adopted a DR for the tropics of 2 (in place of the 1.45 adopted by Keeling et al.^5^), as suggested by the results of Yang et al.^11^, there was no significant change to our results.

For Approach ^#^3, we assumed DR ∝ −MAP, declining from 2.0 at MAP = 1170 mm to 1.0 at MAP = 4000 mm; this reduces the strength of the inferred relationship between *dW/dc*_a_ and MAP.

For Approach ^#^4, we assumed DR ∝ + MAP, increasing from 1.0 at MAP = 1170 mm to 2.0 at MAP = 4000 mm, which enhances the *dW/dc*_a_—MAP relationship. *W* was calculated as: Eq. (3).

3 $$A/g_s = c_{\mathrm{a}} \cdot \left( {1 - c_i/c_{\mathrm{a}}} \right)/1.6$$

Where *A* is net photosynthesis and *g*_s_ is stomatal conductance.

### Statistical tools

We used regression analyses to assess the rate of change in *W* with *c*_a_. Linear mixed models were initially used to test for differences between *W* of Data Set 1 and Data Set 2 for the period 1990–1995. We also used linear mixed models to quantify the combined influences of N-fixing status and *c*_a_ on *W* by testing for differences between the legume and non-legume isotope series presented in van der Sleen et al.^23^. Site was treated as a random variable in all linear mixed model analyses.

All statistical analyses were completed with SPSS or R.

## Supplementary information

Supplementary Information

## Data Availability

All data used in results are available from the first author, as extracted from primary sources or as provided by original authors.

## Code availability

Code used in calculations of model estimates of rates of change is the property of T.N.B. and is available on request (tnbuckley@ucdavis.edu).
