# Supplementary material for: Rainfall drives variation in rates of change in intrinsic water use efficiency of tropical forests
Source: Nat Commun. 2019 Aug 14;10:3661. doi: 10.1038/s41467-019-11679-8 (PMC6694106; doi:10.1038/s41467-019-11679-8)
Supplement: Supplementary file 1 — Supplementary Information [file 41467_2019_11679_MOESM1_ESM.pdf]

Supplementary Information:

Rainfall drives variation in rates of change in intrinsic water use efficiency of tropical forests.

Adams et al.

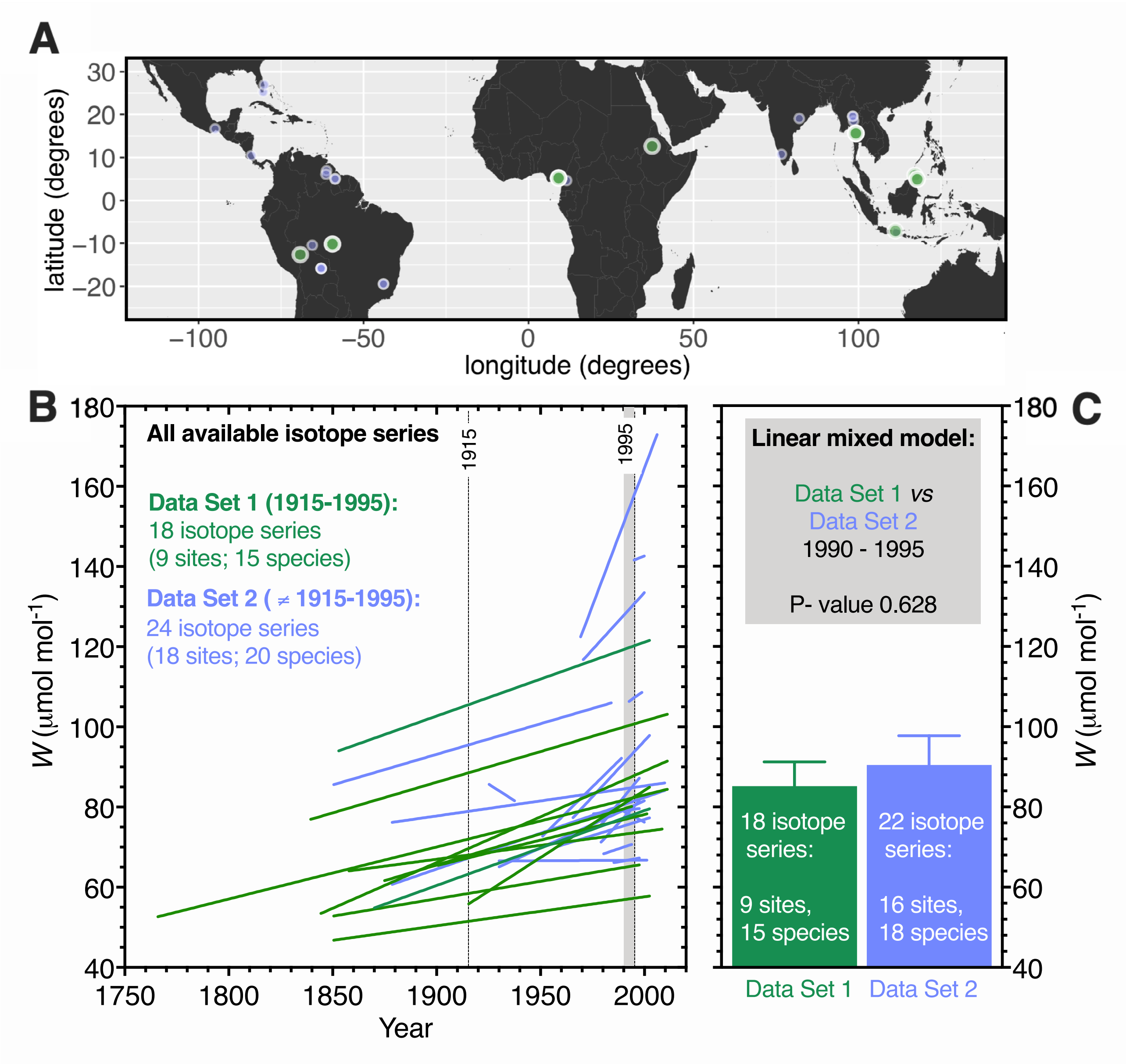

Supplementary Figure 1. Study site locations and comparison of short and long-term *W*.

(A) Locations of sites that provided data used in this study. The figure was prepared using R and public domain packages maps and ggplot2.
(B) Relationships between intrinsic water use efficiency (*W*) and year for all available studies of tropical trees.
(C) Comparison of *W* (mean +/- 1 SE, *P*-value derived from a linear mixed model) for the period 1990-1995 for Data Sets 1 and 2 (See Methods). Green represents Data Set 1, purple represents Data Set 2.

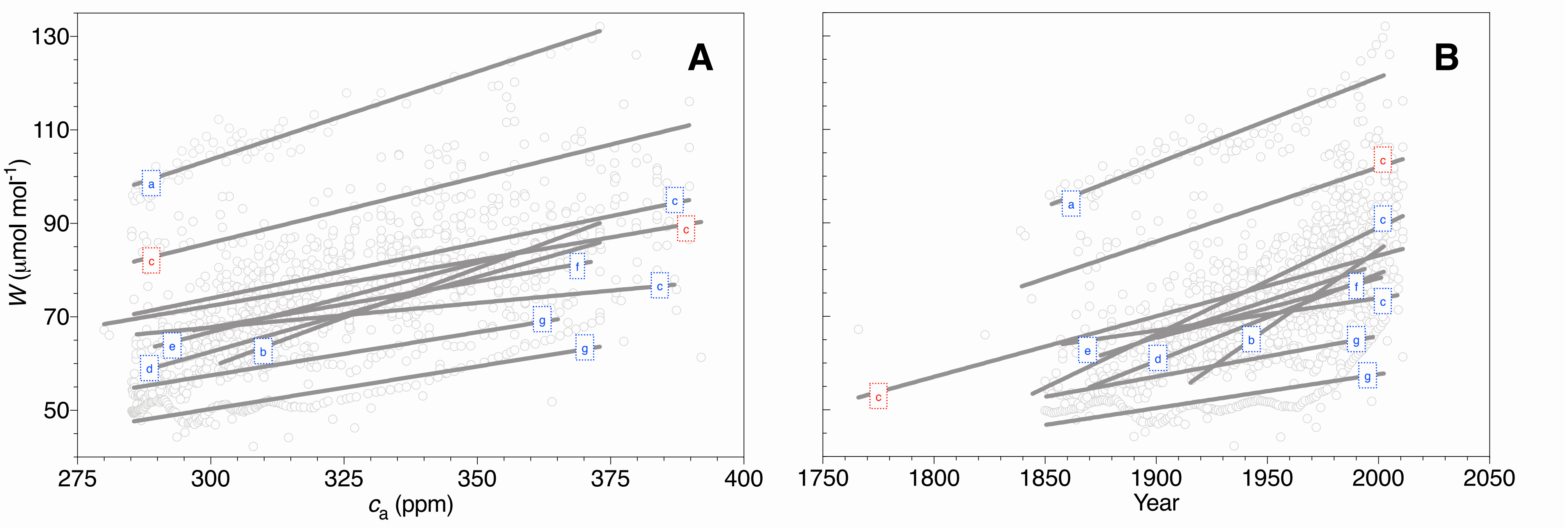

Supplementary Figure 2. Relations of *W* to c_a and year for individual studies.

Relationships between intrinsic water use efficiency (*W*) and (A) atmospheric [CO_2] (c_a), and (B) time. Each long-term isotope series spanned at least the period 1915 – 1995, for tropical biomes. All data are site averages. Relationships marked in red in both (A) and (B) are legumes. For both (A) and (B), data sources are: (a) Wils *et al.*[24], (b) Nock *et al.*[27], (c) van der Sleen *et al.*[23], (d) Ballantyne *et al.*[25] (e) Hietz *et al.*[29] (f) Schollaen *et al.*[28], (g) Loader *et al.*[26] Details of data sources are given in Table 1.

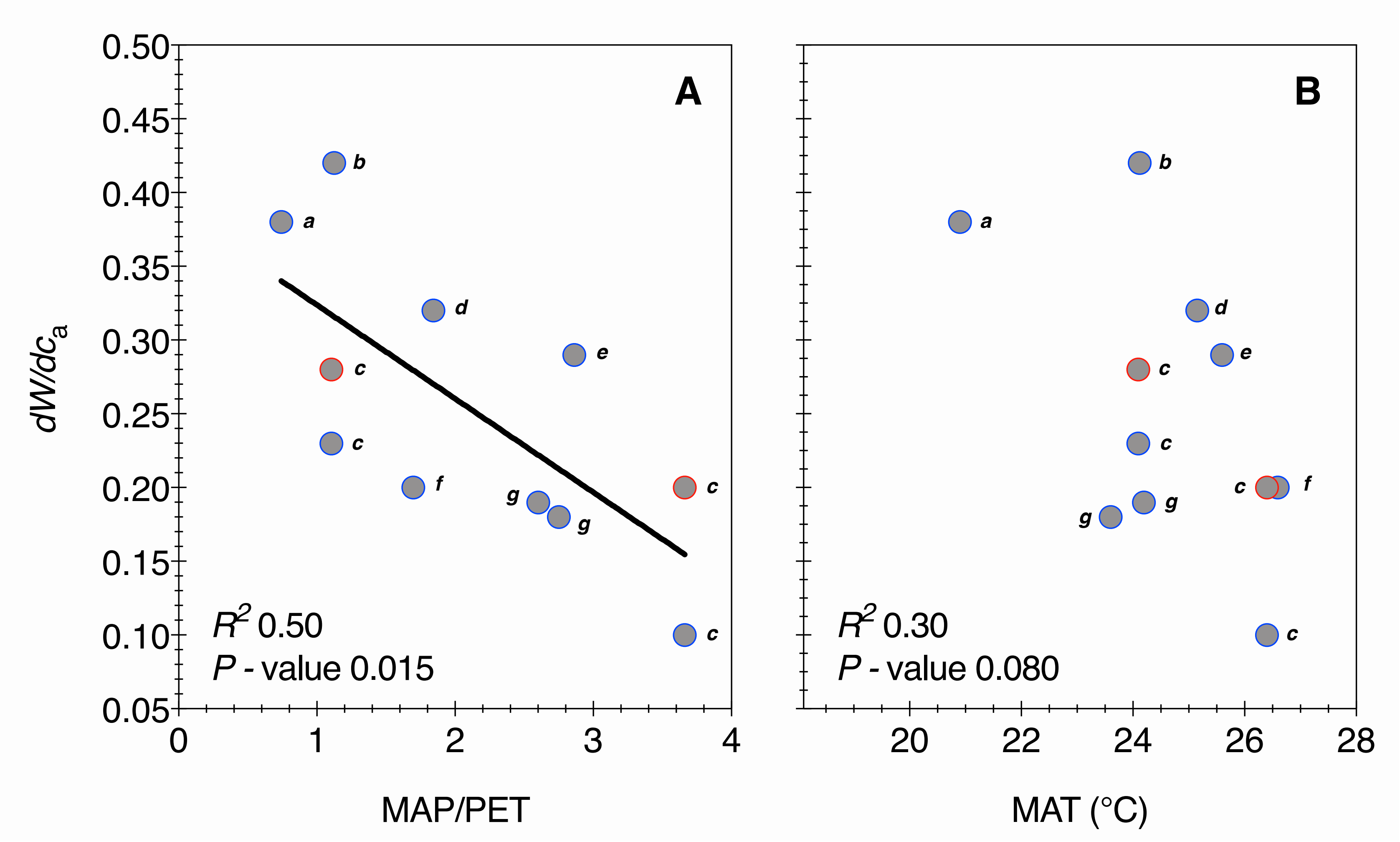

Supplementary Figure 3. Climatic influences on rates of change in *W* with c_a.

(A) Relationship of dW/dc_a (change in intrinsic water use efficiency (W) per unit atmospheric [CO_2] (c_a)), as derived from tree-ring isotope series) to Mean Annual Precipitation (MAP)/Potential Evapotranspiration (PET), (B) Relationship of dW/dc_a to Mean Annual Temperature. Points marked in red in both (A) and (B) are legumes. For both (A) and (B), data sources are: (a) Wils *et al.*[24], (b) Nock *et al.*[27], (c) van der Sleen *et al.*[23], (d) Ballantyne *et al.*[25] (e) Hietz *et al.*[29] (f) Schollaen *et al.*[28], (g) Loader *et al.*[26]

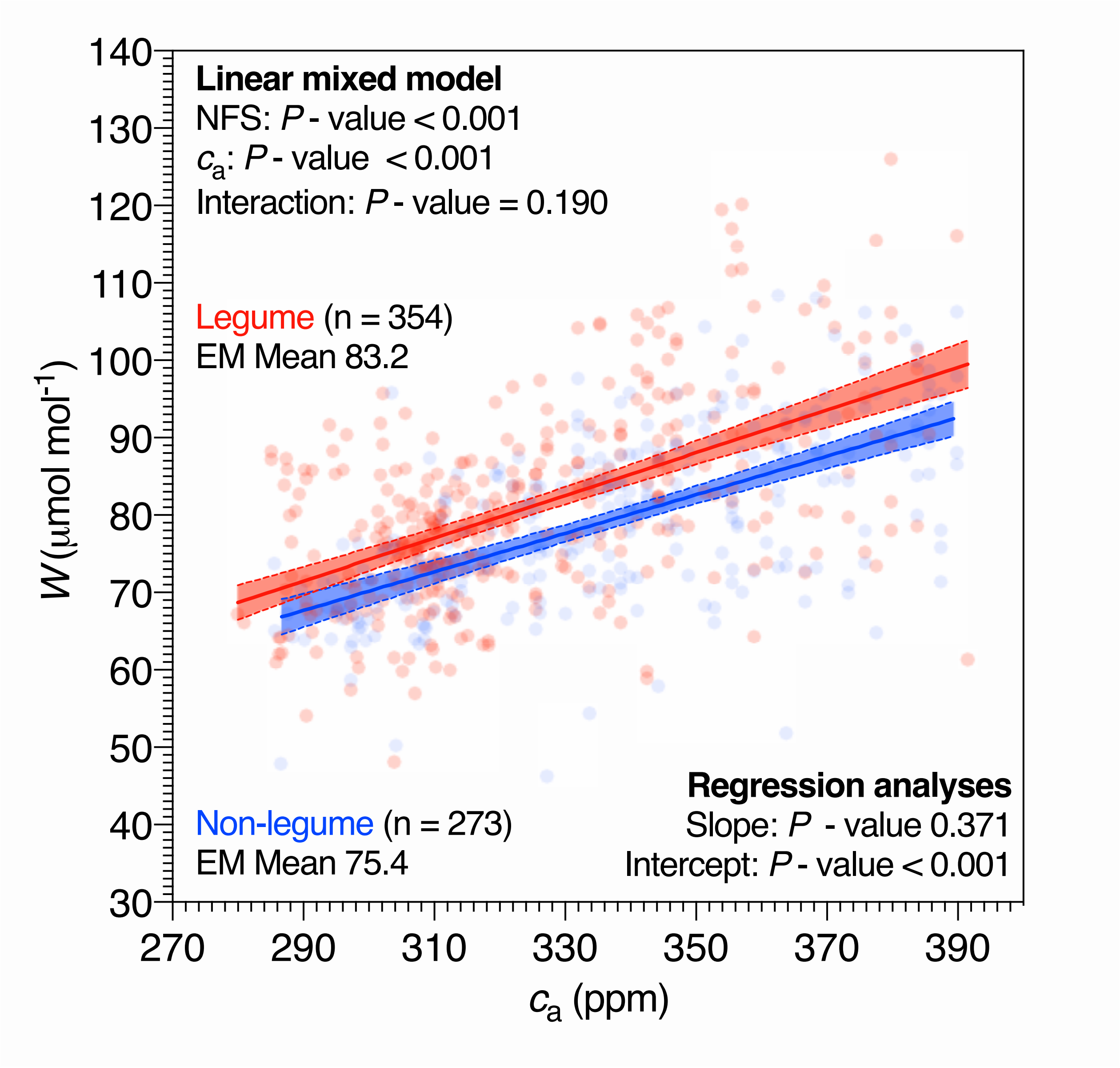

Supplementary Figure 4. Influence of nitrogen fixation on relationship of *W* to c_a.

Relationship between intrinsic water use efficiency *W* (μmol mol^{-1}) and atmospheric [CO_2] (c_a ppm) for sympatric leguminous and non-leguminous trees in tropical biomes between 1850 and 2011. Data shown were sourced from van der Sleen et al.[23]. Linear regressions and 95% CI for the response of *W* to c_a, for 354 individual legumes (red closed circles), and 273 individual non-legumes (blue closed circles) are also shown. Multivariate analyses (linear mixed model) were used to quantify the combined influence of Nitrogen-fixing status (NFS) and c_a on *W* and calculate estimated marginal means (EM Mean) for legumes and non-legumes; bivariate analyses (linear regression) were used to test for differences in slope and intercept of the response of *W* to c_a between legumes and non-legumes.

Supplementary Table 1.

Details of tree species and sites (ranked using rainfall). Wood types are Ring forming (R) or Ringless (RL). Plant groups are Deciduous (D), Evergreen (E) or Semi-deciduous (SD). Legumes are indicated with an asterisk. Response of intrinsic water use efficiency (*W*, calculated from measurements of δ ^{13}C isotope in wood cellulose) and growth to increases in atmospheric $[CO_2]$ (c_a). Data shown is the change in *W* per c_a (dW/dc_a, derived from linear regressions). The significance of relationships between *W* and c_a are denoted as *ns* not significant, ** $P < 0.01$, *** $P < 0.001$.

Species	Wood type	Plant group	Location	MAP	Period	Growth Response	dW/dc_a Response	Data set	Reference
*Mimosa acantholoba**	R	D	Mexico (16°39'N, 95°00'W)	930	1969 - 2007	no response	0.88***	2	Brienen et al.[36]
Podocarpus neriifolius	RL	E	Thailand (18°30'N, 98°30'E)	1000	1993 - 1999		*ns*	2	Poussart et al.[31]
Miliusa velutina	RL	D	Thailand (19°34'N, 98°16'E)	1100	1960 - 1995		*ns*	2	Poussart & Schrag[30]
Quercus kerrii	RL	E	Thailand (19°34'N, 98°16'E)	1100	1980 - 2000		*ns*	2	Poussart & Schrag [30]
Juniperus procera	R	E	Ethiopia (12°37'N, 37°27'E)	1172	1750 - 2003		0.38***	1	Wils et al. [24]
Taxodium ascendens	R	D	USA (25°37'N, 80°24'W)	1432	1970 - 2000		0.38***	2	Anderson et al.[32]
Taxodium distichum	R	D	USA (26°53'N, 80°05'W)	1448	1855-1985		0.38***	NA	Anderson et al.[32]
*Afzelia xylocarpa**	R	D	Thailand (15°36'N,99°12'E)	1473	1838 - 2010	no response	0.28***	1	van der Sleen et al.[23]
Chukrasia tabularis	R	D	Thailand (15°36'N,99°12'E)	1473	1854 - 2008	no response	0.24***	1	van der Sleen et al.[23]
Melia azedarach	R	D	Thailand (15°36'N,99°12'E)	1473	1898 - 2010	negative	0.29***	1	van der Sleen et al.[23]
Toona ciliata	R	D	Thailand (15°36'N, 99°12'E)	1473	1913 - 2011	no response	0.17**	1	van der Sleen et al.[23]
Chukrasia tabularis	R	D	Thailand (15°40'N, 99°10'E)	1500	1915 - 2003	negative	0.41***	1	Nock et al.[27]

Toona ciliata	R	D	Thailand (15°40'N, 99°10'E)	1500	1915 - 2003	negative	0.43***	1	Nock et al.[27]
Melia azedarach	R	D	Thailand (15°40'N, 99°10'E)	1500	1965 - 2003	negative	0.39**	2	Nock et al.[27]
Tectona grandis	R	D	India (19°05'N, 81°57'E)	1557	1929 - 2002		0.43***	2	Managave et al.[33]
Terminalia amazonia	R	E	Venezuela (6°55'N, 61°24'W)	1560	1985 - 1998		*ns*	2	Fichtler et al.[34]
Terminalia superba	R	E	Cameroon (4°40'N, 11°32'E)	1570	1925 - 1938		-1.12*	2	Fichtler et al.[34]
Hura crepitans	R	E	Bolivia (15°50'S, 62°51'W)	1580	1878 - 2008	no response	0.23***	2	van der Sleen et al.[23]
*Sweetia fruticosa**	R	D	Bolivia (15°50'S, 62°51'W)	1580	1878 - 2010	negative	0.20***	2	van der Sleen et al.[23]
Cariniana ianeirensis	R	SD	Bolivia (15°50'S, 62°51'W)	1580	1914 - 2008	no response	0.13***	2	van der Sleen et al.[23]
Ampelocera ruizii	R	E	Bolivia (15°50'S, 62°51'W)	1580	1988 - 2010	no response	*ns*	2	van der Sleen et al.[23]
*Peltogyne cf. heterophylla**	R	SD	Bolivia (10°26'S, 65°33'W)	1690	1994 - 2000	no response	*ns*	2	van der Sleen et al.[38]
Tectona grandis	R	D	Indonesia (7°27'S, 111°33'E)	2100	1950 - 1990		0.48***	2	Poussart et al.[31]
*Samanea saman**	RL	D	Indonesia	2100	1978 - 1998	negative	0.37**	2	Poussart et al.[31]
Tectona grandis	R	D	India (10°46'N, 76°39'E)	2199	1929 - 1990		0.22***	2	Managave et al.[33]
Tectona grandis	R	D	Indonesia (7°05'S,111°11'E)	2200	1900 - 2002		0.20***	1	Schollaen et al.[28]
*Hymenaea stigonocarpa**	R	SD	Brazil (19 27'S, 43 58'W)	2240	1954 - 2005	no response	0.15***	2	Locosselli et al.[34]
*Hymenaea coubaril**	R	SD	Brazil (19 27'S, 43 58'W)	2240	1958 - 2003	no response	0.12**	2	Locosselli et al.[37]

Cedrela odorata	R	D	Peru (12°37'S,69°11'W)	2400	1850 - 2003		0.32***	1	Ballantyne et al.[25]
Goupia glabra	RL	D	Guyana (5°02'N, 58°37'W)	2680	1989 - 2001		*ns*	2	Pons & Helle[35]
Carapa guianensis	RL	SD	Guyana (5°02'N, 58°37'W)	2680	1990 - 2001		-0.63***	2	Pons & Helle[35]
Shorea superba	RL	E	Borneo (4°57'N,117°48'E)	2873	1850 - 2000		0.15***	1	Loader et al.[26]
Shorea johorensis	RL	E	Borneo (4°57'N,117°48'E)	2873	1850 - 2002		0.23***	1	Loader et al.[26]
Terminalia amazonia	R	E	Venezuela (6°06'N, 61°23'W)	2975	1975 - 1998		*ns*	2	Fichtler et al.[34]
Eusideroxylon zwageri	RL	E	Borneo (5°53'N,117°05'E)	3000	1850 - 2003		0.18***	1	Loader et al.[26]
Swietenia macrophylla	R	E	Brazil (10°09'S,59°26'W)	3000	1855 - 1995		0.24***	1	Hietz et al.[29]
Cedrela odorata	R	D	Brazil (10°09'S,59°26'W)	3000	1855 - 1995		0.35***	1	Hietz et al.[29]
*Daniellia ogea**	R	D	Cameroon (5°13'N,9°06'E)	4000	1786 - 2011	no response	0.14***	1	van der Sleen et al.[23]
Terminalia ivorensis	R	E	Cameroon (5°13'N,9°06'E)	4000	1855 - 2009	increase	0.10***	1	van der Sleen et al.[23]
*Brachystegia eurycoma**	R	D	Cameroon (5°13'N,9°06'E)	4000	1894 - 2003	no response	0.21***	1	van der Sleen et al.[23]
*Brachystegia cynometroides**	R	D	Cameroon (5°13'N,9°06'E)	4000	1907 - 2008	negative	0.16***	1	van der Sleen et al.[23]
Terminalia amazonia	R	E	Costa Rica (10°26'N, 83°59'W)	4015	1978 - 1998		0.42***	2	Fichtler et al.[34]
